# Supplementary material for: Drp1 Overexpression Decreases Insulin Content in Pancreatic MIN6 Cells
Source: Int J Mol Sci. 2022 Oct 15;23(20):12338. doi: 10.3390/ijms232012338 (PMC9604375; doi:10.3390/ijms232012338)

Supplementary Data

Drp1 Overexpression Decreases Insulin Content in Pancreatic MIN6 Cells

Uma D. Kabra [1,2], Noah Moruzzi [3], Per-Olof Berggren [3] and Martin Jastroch [4,*]

Supplementary Figure S1: Less exposed immunoblot of OXPHOS complexes of Figure 2E.

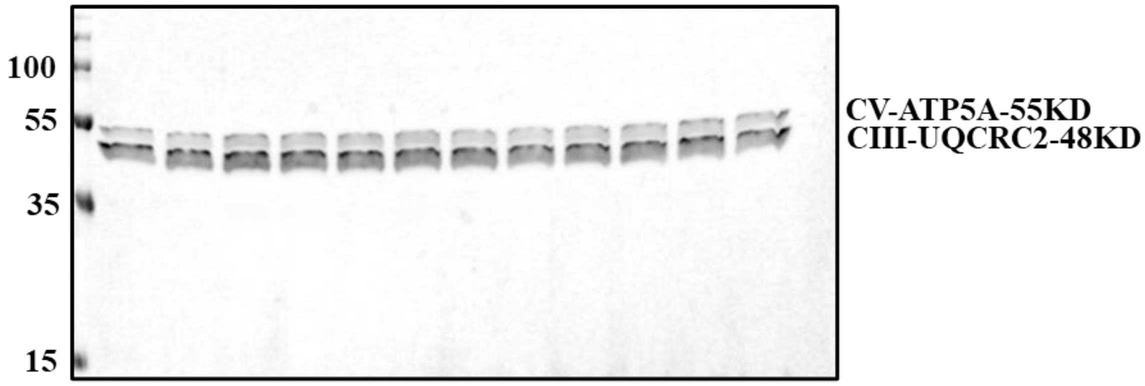

Supplementary Figure S2: Effect of an unrelated protein on insulin content. MIN6 cells were transiently transfected using p-ENTR_RIP-GFP. A. Insulin content expressed at ng of DNA. Data are represented as mean ±SEM (n=4) and n-values represent independent experiments. B. Representative microscopic image of GFP expression.

A

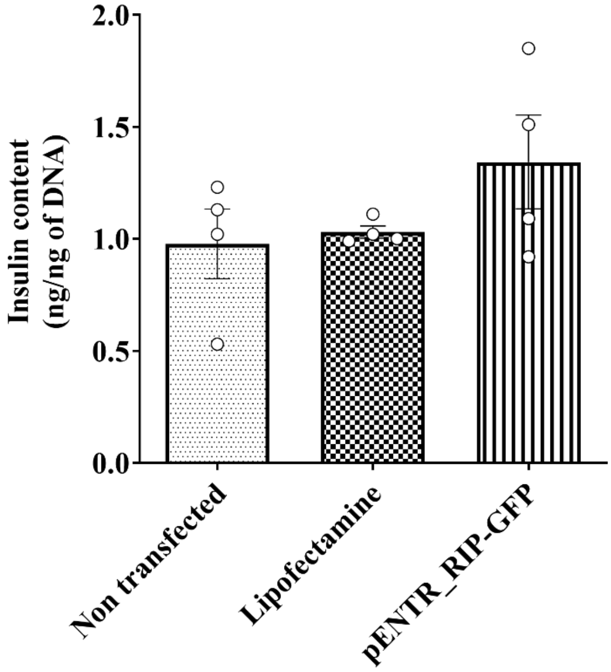

B

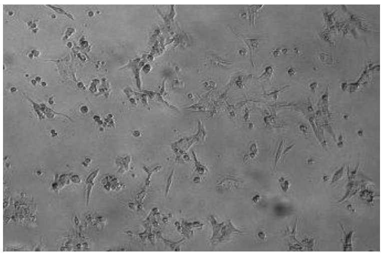

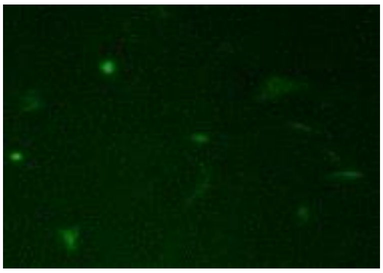

Supplement: Supplementary file 1 [file ijms-23-12338-s001.zip › ijms-1878602-supplementary.pdf]
